# Elements of General and Pathological Anatomy; Presenting a View of the Present State of the Knowledge in These Branches of Science

**Published:** 1852-01

**Authors:** 


*Elements of General and Pathological Anatomy; presenting a view of the present state of the Knowledge in these Branches of Science.* By DAVID CRAGIE, M. D., F. R. S. E., etc. Second edition, enlarged, revised and improved. Philadelphia: Lindsay & Blackiston, 1851. 8 vo. pp. 1072.

The first edition of this work appeared so long ago as 1828. In the present edition, the author states all the materials of the first to have been employed, but "they have been greatly increased by the introduction of new matter under the proper heads, in order to carry forward to the present time the information acquired since the appearance of the first edition." Moreover, "two new books have been added, one on the structure of morbid states of the glands, the other on the structure and morbid states of the lungs and heart."

From the number of pages, it will be perceived that they make a portly volume. It would be worse than affectation for us to imply that we are prepared to speak of its merits from a careful and thorough perusal. We have DIPPED INTO it somewhat, deferring farther examination for a more "convenient season"— a common excuse for postponing bibliographical as well as more solemn duties. From cursory reference to portions of the work, we were led to regret that the author did not prepare an entirely new volume, which should be up to the times, instead of superadding improvements to a treatise adapted to the state of science more than twenty years ago.

